# Electroencephalographic evidence of gray matter lesions among multiple sclerosis patients: A case-control study

**DOI:** 10.1097/MD.0000000000027001

**Published:** 2021-08-20

**Authors:** Ahmed Abduljawad Salim, Safaa Hussain Ali, Ansam Munadel Hussain, Wisam Nabeel Ibrahim

**Affiliations:** a Department of Medicine, College of Medicine, University of Basra, Basra, Iraq; b Department of Physiology, College of Medicine, University of Al-Mustansiriyah, Baghdad, Iraq; c Basra General Hospital, Basra, Iraq; d Department of Biomedical Sciences, College of Health Sciences, QU Health, Qatar University, Doha, Qatar; e Biomedical and Pharmaceutical Research Unit, QU Health, Qatar University, Doha, Qatar.

**Keywords:** alpha rhythm, EDSS, EEG, electroencephalography, expanded disability status scale, MS, multiple sclerosis, PDR, posterior dominant rhythm

## Abstract

This study aimed to investigate evidence of gray matter brain lesions in multiple sclerosis (MS) patients by evaluating the resting state alpha rhythm of brain electrical activity.

The study included 50 patients diagnosed with MS recruited from the MS clinic with 50 age and gender-matched control participants. The study investigated parameters of posterior dominant rhythm (PDR) in the electroencephalography (EEG) recordings including wave frequency and amplitude. Functional disability among the patients was evaluated according to the expanded disability status scale. Univariate statistical analysis was completed using one-way analysis of variance and *t* test with a *P* value of less than .05 to indicate statistical significance.

Patients with MS had significantly lower PDR frequency and amplitude values compared to the controls (*P* value < .01) and 34% of the MS patients had a PDR frequency of less than 8.5 Hz. The PDR frequency was negatively associated with the level of functional disability among the patients (*P* value <.001) and 4% of the patients had abnormal epileptiform discharges.

Background slowing of resting alpha rhythms and epileptiform discharges are suggestive of gray matter degeneration and may help in the prediction and follow-up of cortical damage and functional disabilities among MS patients. Therefore, electroencephalography monitoring of the PDR spectrum may serve as an alternative or complementary tool with other imaging techniques to detect and monitor cerebral cortical lesions.

## 1 Introduction

**Figure 1:**
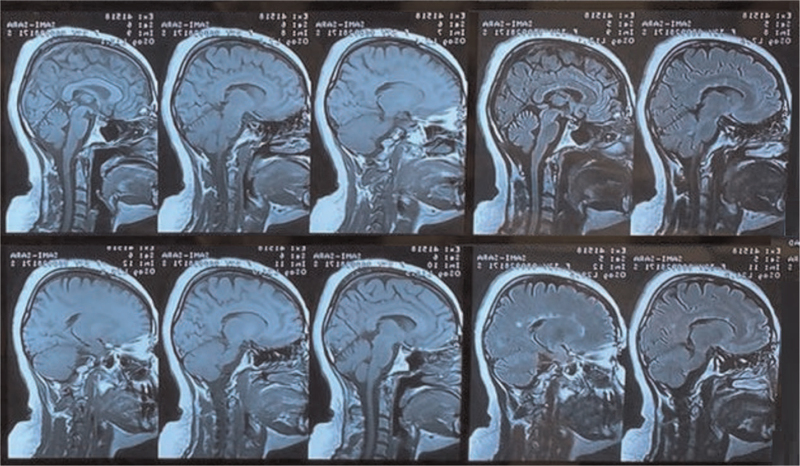
Magnetic resonance imaging demonstrating multiple sagittal section views of a multiple sclerosis patient brain highlighting in black arrows typical multiple white matter plaque lesions in the periventricular region of the brain.

Multiple sclerosis (MS) is a chronic persistent neurological disorder of autoimmune etiology involving the white matter of the central nervous system (CNS).^[1]^ The autoimmune etiology of the disease is still poorly understood particularly the factors that trigger its initiation, however, a growing body of evidence indicates an interplay between genetics and environmental factors such as smoking, vitamin D deficiency, and Epstein–Barr virus infection.^[2]^ The incipient determinants of demyelination in MS involve scattered inflammatory plaques within the white matter of the CNS that progressively lead to functional disabilities. Figure 1 demonstrates the inflammatory plaques of scars in the magnetic resonance imaging (MRI) of MS patients. The characteristic pathological changes in these lesions include inflammation, reactive gliosis, demyelinating, and neuroaxonal degeneration with loss of the myeline-producing oligodendrocyte cells. ^[1,3,4]^

As one of the commonest neurological disorders causing disabilities, MS affects nearly 0.1% of the world's population mainly young females.^[5]^ Patients with MS usually present with acute attacks of neurological relapses due to demyelination. As the disorder progresses, it self-perpetuates and accruals to progressive neuronal degeneration causing lifelong disabilities.^[3,6]^ The course of the diseases may also include intermittent periods of remission with axonal remyelination causing a short-term restoration of the affected brain functions.^[3,6]^

Cortical demyelination is an often overlooked, devastating outcome of the disease which may progress silently over years leading to significant shrinkage of brain tissue.^[2,7–9]^ The onset of cognitive dysfunction may pass unnoticed in the early stages of MS as cerebral lesions progress silently, often difficult to detect by standard neurological examination.^[10]^ As cortical demyelination progresses, it leads to significant cerebral atrophy that is manifested clinically in the form of cognitive impairment and is usually detected in the advanced stage by routine radiological imaging.^[8]^ Memory, learning (especially recall), and abstract reasoning are the earliest involved cognitive functions.^[11]^ As the disease progresses to involve more brain areas, cognitive dysfunction expands to involve linguistic ability, attention, and short-term spatial memory entraining patients to more social disabilities overlaying the physical disabilities of the disease leading to a considerable decrease in the quality of life.^[11]^

Electroencephalogram (EEG) represents a promising tool that may help in the early detection and monitoring of cognitive impairment among MS patients.^[12]^ The changes in brain electrical activity in EEG may detect deteriorations of cerebral neurons in the form of slowing in the background activity.^[13]^ The background brain activity is generally indicated by the alpha rhythm which is represented by the waves depicted during relaxed wakefulness with the eyes closed.^[13]^ This rhythm is mostly detected over the posterior region of the scalp and is commonly referred to as the posterior dominant rhythm (PDR). The origin of PDR is not well identified but it may represent complex interactions between cortical neurons, pyramidal cells with the involvement of the thalamic input.^[13]^

The study aimed to assess alterations of background brain wave frequencies (ie, electrocortical) as an indicator of underlying cortical lesions in MS patients using EEG recording. This approach may help in the early detection of cortical brain lesions in MS patients before the development of clinical manifestations underlined by extensive cortical lesions.

## 2 Materials and methods

### 2.1 Study characteristics

The study was conducted in the Multiple Sclerosis Clinic of Basra General Hospital, Basra, Iraq. The study protocol and participant questionnaires were approved by the institutional ethical committee in the college of medicine, Basra University before starting the study. The case-control study included 50 MS patients who were diagnosed by 5 neurologists in the MS clinic per the 2010 Revised McDonald criteria.^[14]^ The patients were in remission for at least 1 month and were conveniently selected from the center during their regular visits for evaluation and treatment. Patients with MS were excluded from the study if they presented with epilepsy syndrome, epilepsy secondary to other conditions like head trauma, and patients with early-onset neurodegenerative diseases. The control participants were symptom-free volunteers with no history of MS or any neurological diseases that may affect EEG recording and were matched in age and gender with the patients. All study participants consented to the study by signing the forms before the study was conducted. This was followed by filling a survey questionnaire in which the responses provided information about participants’ demographic characteristics, duration and type of disease, medications, family history, and past medical history.

### 2.2 Electroencephalography

The electrocortical activity of the participants was examined using the BrainQuick electroencephalograph instrument (Micromed SpA, Treviso, Italy) analyzed with System Plus Evolution software version 1.04 (Micromed SpA, Treviso, Italy). Twenty-one scalp electrodes (Micromed SpA, Treviso, Italy) were placed according to the International 10–20 System for electrode placement. Ac cream paste was used to improve the conduction of scalp electrodes (Ac cream, Spes Medica S.r.l., Genova, Italy). In the study, the EEG recording was set in a dark quiet room with participants sitting upright awake with eyes closed for 10 minutes. This was followed by hyperventilation for 3 minutes after exclusion of cardiac or respiratory diseases ending with flash stimulation for 30 seconds.^[15]^ during the recording, all the study participants were awake and none of the participants were drowsy or asleep. The EEG recordings were performed with typical longitudinal bipolar montage with a visual display of 30 mm/s, filter settings were 1 Hz for low pass frequencies and 70 Hz for high pass frequencies with a notch filter of 60 Hz. The amplifier sensitivity was calibrated within the range of 20 to 100 μv/cm to optimize the visualization of brain waves activity. None of the calibration measures affected the EEG parameters of amplitude or frequency. The background activity was highly regular and multiple artifacts-free epochs were selected throughout the recording at different stages and time intervals to calculate the mean frequency and amplitude of brain wave activity to ensure the accuracy of the measured values. The frequency was measured using fast Fourier transform power which reformats data into component frequencies.

### 2.3 The expanded disability status scale

The expanded disability status scale (EDSS) is a clinical scale that is used by neurologists to assess functional disabilities among MS patients. The scale is based on the clinical observation of neurological deficits in 8 functional domains including pyramidal, cerebellar, brainstem, sensory, bowel and bladder, visual, and cerebral functions. The scale ranges from 0 to 10 with 0 indicating no disability and 10 indicating death due to MS.^[16]^ This scale is commonly used to assess the level of neurological deficits and to follow up the progression of symptoms among MS patients.

### 2.4 Statistical analysis

Demographic and EEG parameters (PDR amplitude and frequency) were presented in tables as frequencies for categorical variables and mean values with standard deviation (SD) for numerical variables. Statistical analysis was completed using GraphPad Prism version 8 for Windows, (GraphPad Software, La Jolla California USA). Parametric tests were used based on D’Agostino–Pearson omnibus normality test. An independent *t* test was used to assess the association of EEG parameters (PDR amplitude and frequency) with MS disease status. To confirm the effect of the progressive course of the disease and the precipitating neurological deficits, one-way analysis of variance test was used to assess the association between the PDR parameters and the duration of MS disease or EDSS score. A *P* value of <.05 was used to indicate statistical significance.

## 3 Results

### 3.1 Baseline demographic and clinical data

The demographic and clinical characteristics of the study participants are demonstrated in Table 1. Most of the participants aged below 40 years with an average of 35 and 37 years for patients and controls, respectively. Most of the patients were of female gender (68%) and the main type of clinical presentation was the relapsing-remitting MS (RRMS) followed by primary progressive MS (PPMS) and secondary progressive MS (SPMS) types respectively with disease duration less than 10 years in the majority of the patients. The patients were treated with Betaferon, Rebif, and Avonex (45%, 39%, and 12%, respectively); all 3 medications are different brands of the beta 1-a class of interferons commonly used in the treatment of MS.

**Table 1:** Clinical and demographic characteristics of study participants.

Variables	Patients N (%)	Controls N (%)
Age (yrs)		
<40	33 (66)	35 (70)
>40	17 (34)	15 (30)
Mean ± SD	37.2 ± 9.731	35.34 ± 9.32
Gender		
Male	16 (32)	16 (32)
Female	34 (68)	34 (68)
Medications		
Betaferon	22 (45)	–
Rebif	19 (39)	–
Avonex	6 (12)	–
No treatment	2 (4)	–
Family history		
Positive	1 (2)	0
Negative	49 (98)	50 (100)
Duration of illness		
<5 years	25 (50)	–
5–10 years	23 (46)	–
>10 years	2 (4)	–
Subtype of MS disease		
RRMS	46 (92)	–
PPMS	2 (4)	–
SPMS	2 (4)	–
PRMS	0	–
EDSS		
0	10 (20)	50 (100)
1	9 (18)	0
2	8 (16)	0
3	3 (6)	0
4	8 (16)	0
5	3 (6)	0
6	2 (4)	0
7	5 (10)	0
8	2 (4)	0

EDSS = the expanded disability status scale, MS = multiple sclerosis, PPMS = primary progressive multiple sclerosis, PRMS = progressive relapsing multiple sclerosis, RRMS = relapsing-remitting multiple clerosis, SD = standard deviation, SPMS = secondary progressive multiple sclerosis.

### 3.2 PDR frequency and amplitude

One of the significant findings in the study was the abnormally low PDR frequency where 17 MS patients (28%) had an abnormal PDR with a frequency of less than 8 Hz. Such observation was not elicited among the control participants. As shown in Table 2, the mean value of PDR frequency was significantly lower among MS patients compared to the control group (*P* value ≤.001) confirming a significant background slowing.

**Table 2:** Analysis of PDR waves frequency and amplitude of the EEG recordings among MS patients and controls.

PDR parameters	MS patients	Controls	*P* value
PDR frequency (Mean ± SD)	8.680 ± 1.587	10.307 ± 0.979	.000^∗∗^
Amplitude of PDR (Mean ± SD)	41.060 ± 20.693	51.618 ± 21.226	.013^∗^

Unite of measurement for PDR waves frequency is Hertz (Hz) while the measurement unites of PDR waves amplitude is μ volt (μv). Data shown are mean value ± SD (n = 50, *t* test, ^∗^*P* value ≤.05, ^∗∗^*P* value ≤.001(. EEG = electroencephalography, MS = multiple sclerosis, PDR = posterior dominant rhythm, SD = standard deviation.

**Figure 2:**
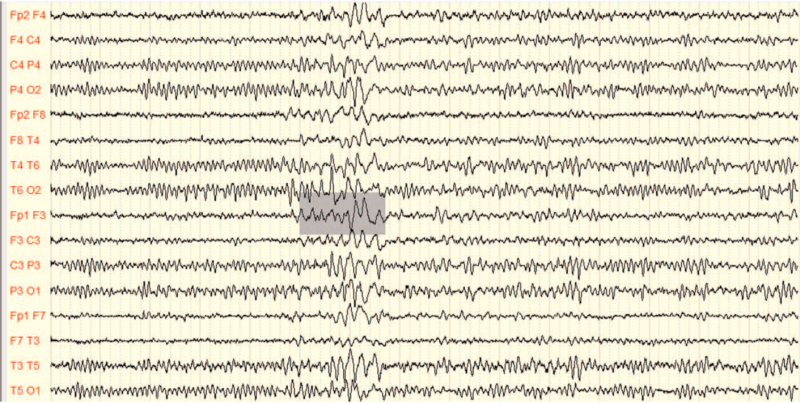
EEG recording of MS patient demonstrating epileptiform activity. Recorded by Micro med Electroencephalograph instrument with System Plus Evolution software. EEG = electroencephalography, MS = multiple sclerosis.

A less significant decrease in the PDR waves amplitude was elicited among MS patients compared to the control group as shown in Table 2 (*P* value = .013). The patients had a mean amplitude of 41.06 μV; while among the controls, it was 51.618 ± 21.226 μV. Interestingly, abnormal epileptiform discharges were detected in 2 patients in the form of focal sharp wave activities as depicted in the EEG recording shown in Figure 2. Such discharge was absent among all control participants.

### 3.3 The expanded disability status scale

**Figure 3:**
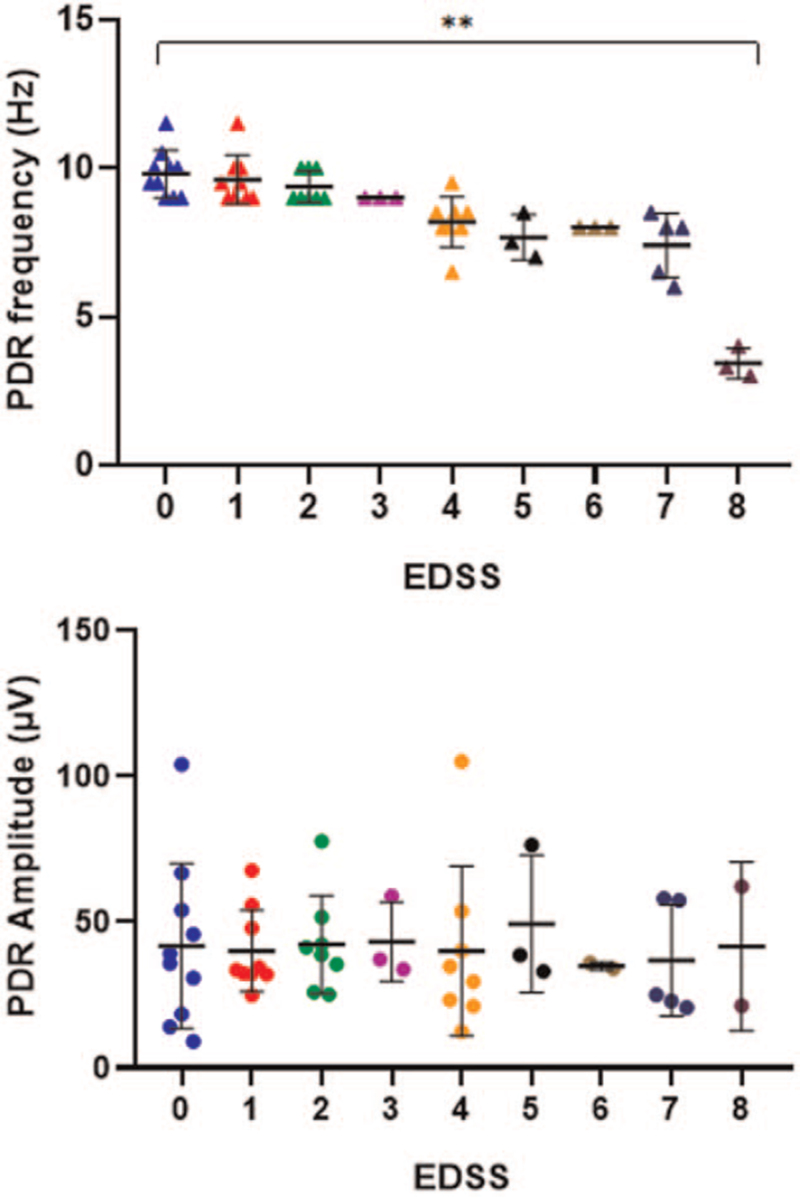
Scatter plot of PDR waves frequencies and amplitudes according to the EDSS among MS patients. Triangle dots are actual PDR frequency values with the horizontal and vertical lines representative of mean and standard deviation values, respectively, one-way analysis of variance test, ^∗∗^*P* value <.00 (. EDSS = expanded disability status scale, MS = multiple sclerosis, PDR = posterior dominant rhythm.

A significant increase in the rate of functional disability was associated with slowing of the PDR frequency as shown in Figure 2. The highest rate of disability was 8 which indicates a significant impairment in the ability to walk was associated with an abnormally low PDR frequency of (3.15 ± 0.22). The amplitudes of PDR waves were not associated with EDSS as shown in Figure 3.

### 3.4 Relation of PDR Frequency or amplitude with the duration of the disease

**Figure 4:**
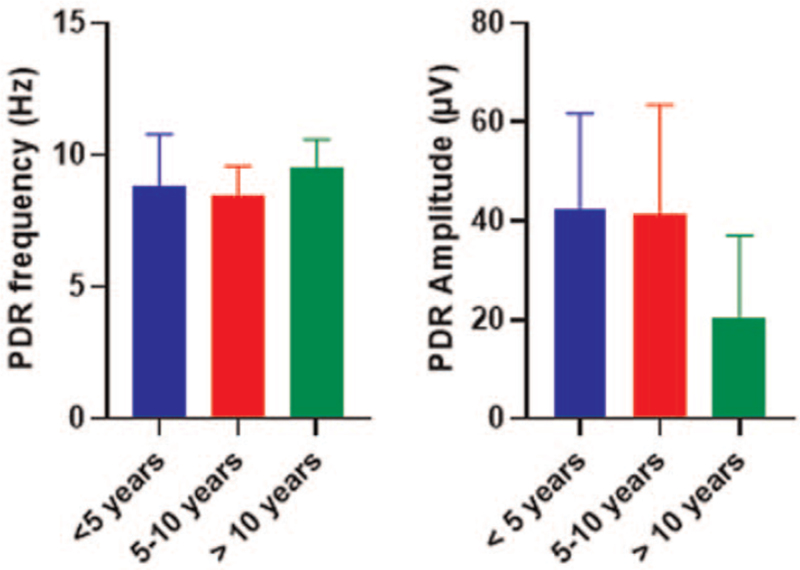
Comparison of PDR wave frequencies and amplitudes distribution among different groups of MS patients according to the disease duration. Data shown are mean value ± SD comparison between groups was completed using one-way analysis of variance test. MS = multiple sclerosis, PDR = posterior dominant rhythm, SD = standard deviation.

The amplitude and frequency of the background PDR were not significantly associated with the duration of MS disease as shown in Figure 4.

## 4 Discussion

The study aimed to evaluate the alpha rhythm spectrum using PDR background activity among MS patients. Several criteria were followed to validate the PDR spectrum in this study including lateral symmetry in amplitudes, continuation, lack of temporary attenuation, spontaneous variability, and reactivity to stimulation or eye-opening.^[13]^ The study participants were kept alert during the EEG recording to rule out the hypnotic influence on the spectral analysis.

The PDR brain activity may vary according to age with its peak frequency of (3–4 Hz) during childhood that increases toward maturation with a peak frequency in adulthood of (8–12 Hz).^[17]^ Although slow oscillations in the PDR or other wave frequencies may indicate nonspecific changes of diffuse encephalopathy; however, these oscillations are commonly used in the evaluation of cognitive functions such as visual perception. The PDR spectrum may also help in the detection of hidden brain pathologies in psychological disorders such as depression and schizophrenia and neurodegenerative disorders such as Alzheimer disease.^[12,13]^ Therefore, these slow oscillations may denote critical and subcortical lesions and may help in predicting cognitive dysfunction among MS patients.

The study confirmed lower PDR frequency among MS patients compared with the controls. Indeed, the PDR represents a valid predictor for background neuronal activity that is usually presented within the alpha frequency indicating a state of cognitive preparedness.^[18,19]^ The PDR frequency range is between 8.5 and 11 Hz; a narrower range compared to the alpha rhythm that may indicate abnormal slowing with PDR waves if the frequencies are lower than 8.5 Hz.^[20]^ Based on functional MRI, the PDR spectrum is considered as an idling rhythm that reflects a decrease in visual processing or conscious attention and is inversely proportional to the occipital cerebral metabolism.^[21]^ the thalamus may also be involved in generating the PDR wave spectrum within thalamic cortical neuronal circuits.^[22]^ The slowing of background neuronal activity may also happen in the form of an increase of delta and theta wave frequencies in the tempero-frontal cerebral region that correlates with memory deficits among MS patients.^[23,24]^ Notably, these slow frequencies are more frequent with high subcortical lesion load than low subcortical lesion load or normal conditions. Therefore, based on the EEG recording and functional imaging, any slowing in the PDR spectrum may indicate pathology within the cortical or subcortical structures due to the accumulated neuronal degeneration within inflammatory plaques among MS patients.^[19]^

Although the PDR amplitude measures are within the normal range except for 2 cases of MS patients who had an amplitude of less than 20 μV; however, these differences were statistically significant (*P* value ≤.05). The low PDR wave amplitude indicates a decrease in the voltage generated by the neuronal activity reaching the scalp electrodes. Based on functional imaging in other studies, the amplitude or power of the PDR spectrum correlates positively with the function of the brainstem, limbic system, and the mesencephalic – medial thalamic regions.^[25]^ Thus, PDR amplitude recession may be attributed to the loss of cortical neurons and cerebral atrophy in these brain locations.

The course of the disease is generally categorized into 4 different types with the most common presentation in the form of relapse of an acute or subacute neurological dysfunction lasting for a minimum of 24 hours usually evolving over days or weeks followed by considerable remission. This presentation is referred to as RRMS.^[26]^ If the RRMS progresses gradually with irreversible neurological deficits and disability, then the type of manifestation is SPMS.^[14]^ A 10% to 15% of patients with MS have insidious disease progression from the onset resulting in the gradual accumulation of neurological deficits, a type of presentation known as PPMS.^[14]^ A rare type of presentation known as the progressive relapsing MS is characterized by having a progressive course from disease onset with superimposed relapses.^[14]^ The participant clinical and demographic characteristics in the study were consistent with global propensity features of MS in which the majority of the patients were females and the commonest subtype of the presentation was RRMS (94% of MS patients) followed by PPMS and SPMS, respectively.^[27,28]^

The disease duration was not associated with slowing of PDR frequency or reduction in PDR amplitude as shown in Figure 4. Many reports highlighted the progressive nature of cortical lesions in MS. This observation may be attributed to the study design. Patients manifested different types of presentations that may not necessarily correlate with the degree of lesions or disabilities. Therefore, a longitudinal study design would be more appropriate to follow up with the patients and confirm the relation of disease duration with changes of PDR parameters. The nonsignificant association may also indicate the involvement of the cerebral cortical-subcortical regions in the early stages before the development of MS symptoms. At the time of symptoms development, the gray matter lesions are probably in the late stage and may have a weak correlation with disease duration.^[8]^

Therefore, correlation analysis was determined between PDR spectral changes and functional disability according to the EDSS system. In this approach, a negative association was evident between the level of disability and the PDR frequency as indicated in Figure 3. More disabilities were evident among patients with PDR frequencies lower than 4 Hz. Functional disabilities partly include the cognitive domain that is negatively associated with the progression of MS disease as indicated by functional imaging techniques.^[29]^ Therefore, the PDR frequency correlates with cognitive function and speed of processing as indicators of cortical and subcortical lesions among MS patients.

Another evidence of cortical degeneration among the MS patients in this study included the detection of epileptiform wave spikes in 2 MS patients as shown in Figure 2. Cerebral cortical lesions may increase the risk of epilepsy among MS patients.^[30]^ In these patients, the epileptic fits tend to occur in the form of generalized or focal seizures with or without secondary generalization.^[30]^ The underlying cause is attributed to the hyperexcitability caused by inflammation, demyelination, and edema in the cerebral cortical-subcortical lesions.^[31,32]^ Indeed, MS patients are prone to have more frequent attacks of seizers than patients with chronic epilepsy due to the influence of chronic cortical plaques that progress leading to permanent alteration of cortical neuron function.^[33]^ This finding is in agreement with Marrie et al who reported the occurrence of seizure disorder among MS patients with an incidence of 2.28% and prevalence of about 3.09%.^[31]^ As indicated by Kelley and Rodriguez in 2009, an increase in seizure incidence in MS patients is about 2- to 3-folds more compared to the general age-matched healthy population due to cortical inflammatory lesions of the disorder.^[34]^

None of the MS patients in this study had a family history of the disease which is probably attributed to the small sample size of patients in the study. Genetic elements contribute to the etiology of MS and its segregation in families. However, the majority of MS cases are sporadic with proximately 20% of precipitation among families that may also share the influence of environmental factors.^[35]^

The use of EEG may help in the detection of early gray matter lesions in MS patients that are obscure by the clinical examination or regular diagnostic imaging techniques. The use of EEG offers improved temporal resolution compared with other imaging techniques such as MRI. However, its limited spatial resolution in precisely allocating the lesion might suggest its use as a complementary tool. Thus, EEG would help in monitoring the progression of cortical degeneration and response to treatment among MS patients due to its ease of use, portability, and low cost.

Limitations of the study include the cross-sectional design that may not accurately reflect the correlations between the PDR spectrum and the progression of MS. Therefore longitudinal studies are more suitable. Another limitation is the relatively small sample size of patients that may affect concluded observations.

## 5 Conclusion

The study confirmed the presence of EEG background waves slowing among MS patients and its correlation with the functional disability of the patients indicating cortical and subcortical neuronal loss among MS patients. Therefore, the use of EEG recording may help as an affordable screening tool in MS patients to indicate the early gray matter lesions that may pave the way for more preventive and therapeutic options for extensive brain atrophy.

## Author contributions

**Conceptualization:** Ahmed Abduljawad Salim, Safaa Hussain Ali.

**Data curation:** Wisam Nabeel Ibrahim.

**Formal analysis:** Wisam Nabeel Ibrahim.

**Funding acquisition:** Wisam Nabeel Ibrahim.

**Investigation:** Safaa Hussain Ali.

**Methodology:** Ansam Munadel Hussain.

**Project administration:** Ahmed Abduljawad Salim.

**Supervision:** Ahmed Abduljawad Salim.

**Validation:** Ahmed Abduljawad Salim.

**Writing – original draft:** Wisam Nabeel Ibrahim.

**Writing – review & editing:** Wisam Nabeel Ibrahim.
